# Hospital and Institutional News

**Published:** 1920-02-21

**Authors:** 


### HOSPITAL AND INSTITUTIONAL NEWS.

### A GUEST OF HONOUR.

The Rt. Hon. C. Addison, M.D., M.P., Sir Robert Morant, K.C.B., and other distinguished guests have intimated their intention of being present at the dinner which the Federation of Medical and Allied Societies is giving, on the 25th inst., at the Café Royal, and at which Lord Dawson of Penn, G.C.V.O., C.B., M.D., is to be the guest of honour.

### THE NEW CONSULTATIVE COUNCIL.

The Consultative Council on General Health Questions, established under the Ministry of Health Act, has been appointed, and held its first meeting on Thursday. The Minister of Health, Dr. Addison, who was accompanied by Lord Astor, Parliamentary Secretary to the Ministry, presided, and asked the Council to put before him a statement of the main defects in existing provisions for safeguarding the health of the people, and to suggest remedies from the standpoint of persons who as members of the general public will be affected by the health services supervised by the Ministry. The members of the Council have been appointed with this special purpose in view. Women form a majority of the membership. Lady Rhondda will act as Chairman and Mr. Arthur Greenwood as Vice-Chairman. The other members are as follows:—Mrs. Aspinall (United Textile Factory Workers' Association), Councillor C. Aveling (Past President of the National Chamber of Trade), Mrs. F. Harrison Bell (Labour Party), Mrs. Burke (Women's Co-operative Guild), Mr. George Goodenough (Parliamentary Committee of the Co-operative Congress), Mrs. Ogilvie Gorden, D.Sc. (President of the National Council of Women of Great Britain and Ireland), Mr. W. L. Hichens (Chairman of Messrs. Cammell, Laird & Co., Ltd.), Mrs. Hood (Women's Co-operative Guild), Mr. W. Littler (Civil Service Alliance), Mr. Samuel Lord, F.S.S. (National Association of Local Government Officers), Miss Margaret Macmillan (Labour Party), Mrs. Mayo (a member of the Dorsetshire County Council with a wide knowledge of rural conditions), Mr. F. H. Norman (Professional Workers' Federation), Miss E. M. Phelp (National Association of Domestic Workers), Mrs. Pember Reeves, Lady Edmund Talbot, and Miss Gertrude M. Tuckwell, J.P.

### LONG-STANDING MILITARY CASES.

We learn from *The Times* that, owing to the fact that many long-standing medical and surgical cases of wartime origin have remained in the military hospitals beyond the limit of time in which benefit is probably to be derived, the medical authorities of the London district have assembled a board of experienced consultants systematically to go into the question. Hospitals are to be visited and advice given in the interests of both the patients and the State as to the best means to be adopted for the men's recovery and disposal. The names given are Brevet-Col. H. J. Waring, R.A.M.C. (T.), president, Brevet-Col. F. McLennan, R.A.M.C., Major W. E. Wynter, R.A.M.C. (T.), Capt. C. M. Hinds Howell, R.A.M.C. (T.), and Col. T. H. Openshaw (for orthopædic cases when required). This is not a step, as the patient might at first sight think, simply towards getting rid of him as quickly as possible. The extremely fair dealings of the pensions boards, and in fact all organisations controlling the welfare of our disabled men, have amply proved the absence of this tendency in official circles. The fact is that as time goes on the cases still left in their charge become by a natural process of elimination those of the more complex and difficult types, and hence the appointment of this and the other advisory boards which have recently come into existence is not only necessary but thoroughly to be commended.

### MENTAL DISORDERS.

With reference to our contributor's article on incipient mental disease, appearing on page 483, we would call attention to the letter in *The Times* of February 12 from Professor Robertson, of Edinburgh University. His words come in reply to the letter on the same subject calling for reforms in the English procedure with this class of case, and the chief points he would have us note are as follows: In Scotland mentally deranged patients who have means, or whose friends can pay for their support, are able at the present time to receive curative treatment in any house or home without being certified as insane and without being sent to an asylum. This principle has worked satisfactorily for half a century, and so may presumably be recommended for adoption in this country, but the financial problems connected with such reform are not so simple. The difficulty here lies in the fact that in Scotland a grant is given by the State for the maintenance of any person who is certified to be a lunatic, but this grant is withheld, although the patient be treated on similar medical lines, if he or she be not *certified* insane. The resulting effect is an inevitable reduction in practice of the good to be expected to the first-mentioned freedom given by the more considerate system in Scotland.

### GRANTS FOR THE BLIND.

The Ministry of Health are now making grants in aid of the blind, and, with a view to co-ordination of effort, have secured the close co-operation of the National Institute for the Blind, of which Sir Arthur Pearson is President. A Joint Committee, representing the Advisory Committee on the Welfare of the Blind, which sits at the Ministry, and the National Institute for the Blind, has accordingly been set up to advise on matters which jointly concern them, and a desire is expressed that ways of diminishing overlapping and of preventing waste of effort in the collection of voluntary contributions shall, among other things, be considered.

### A WORKING MAN'S LEGACY.

It is always a pleasure to record a legacy to hospitals from a working man; first, in that he was able to make it; secondly, that he gave it to a hospital. King Edward VII. Hospital, Cardiff, has received an account of such a legacy, £350 from the estate of the late Mr. Thomas Williams, a worker on the Taff Vale Railway, who had once been a patient in the hospital. The gift is to be commemorated by a tablet to his memory.

### POLITICS AND HOSPITAL APPOINTMENTS.

In a recent speech in favour of the amalgamation of Bristol hospitals, Mr. Hey Groves made some interesting remarks on the influence of politics upon hospital appointments. Our readers will be relieved to hear that the Bristol surgeon was speaking of the past. He declared that Bristol Royal Infirmary started as a representative of Conservative politics, and that the General Hospital was founded "on the Liberal side," and that within living memory appointments were made partly on political grounds. The war, he said, had ended party warfare, and the hospitals too should abolish jealousy and rivalry, and substitute co-operation in their room. The argument tends (not to the foundation of a new Coalition hospital), but to amalgamation, which, Mr. Hey Groves says, will not only reduce expenses, but also improve the work of the special departments, which need in one place the staff and equipment now divided between two institutions or more. He deplores the multiplication of new hospitals by private generosity, and says that no new hospital should be founded without a licence.

### MILITARY FURNITURE AND AMBULANCES.

Some dismay has been expressed in various districts at the decision of the War Office to remove the furniture from military hospitals, and not to sell it in the districts where they were situated, thus depriving local hospitals and purchasers of any opportunity to buy. It is said that the furniture in some cases has been sent to the ravaged districts of France, and, if that is true, we suppose that nothing more can be said. But a timely question is the destination of the Army ambulances, which many hospitals would be glad to have an opportunity to purchase. On this no information has been offered. We should like to know what has happened to them.

### THE KITCHENER MEMORIAL IN EGYPT.

The endowment of a hospital and medical college for women is part of the proposed scheme for a Kitchener Memorial in Egypt. According to the Cairo correspondent of *The Times*, funds have not yet been subscribed, but it is suggested that the Red Cross Society should make a grant from its surplus funds for this purpose.

### A LIMB-FITTING HOSPITAL FOR CIVILIANS.

In accordance with the promise made when the institution was founded, the Prince of Wales's Hospital for the Limbless at Cardiff will ere long be devoted to the interests of civilian patients. The fitting of limbs for sailors and soldiers is almost a completed task, but in Wales there exists a larger number of limbless civilians than naval and military men. At a meeting of the Council held last week, over which Mrs. Lloyd George presided, steps were taken to reconstitute the governing body in accord with the altered circumstances. The Navy and Army representatives are to be eliminated, and in their place delegates of certain industrial and other interests will be appointed. Some idea of the work done at the hospital may be gathered from the fact that in six months last year ending in November 2,292 limbs and appliances were fitted.

### CARDIFF'S GREAT EFFORT.

Thursday of last week marked the close of the first month of the Lord Mayor of Cardiff's appeal for £150,000 for King Edward VII. Hospital, and Mr. Leonard Rea, the secretary, was able to report at the board meeting on that day that £93,818 had been subscribed, and that if the fund reached £95,000 on the morrow, as it probably would, it constituted, he believed, a hospital record for the whole Kingdom. The raising of all but £100,000 for a hospital within the space of a month is a wonderful achievement by Cardiff's Lord Mayor, whose year of office is being made memorable by his telling advocacy of the institution's needs and the solidly practical results accruing therefrom. Among the latest subscriptions is one of £1,050 from the Ocean Steam Coal Company, Ltd., who accompanied their generous cheque with an intimation that they intended in future to contribute £200 a year to the hospital funds.

### THE CASE FOR LAMBETH INFIRMARY.

Dr. Lionel Baly, the medical superintendent at Lambeth Infirmary, in a report to his infirmary committee, points out that it is of the utmost importance that local practitioners and the public should know that general hospitals cannot be relied upon to admit cases of extreme urgency, and it was therefore a waste of valuable time in such cases to make application unless it was made quickly by telephone. He had frequently advocated the necessity for the guardians to be prepared to deal with every emergency. Only one case of an urgent nature in twelve years had failed to secure treatment at the infirmary, and in that case they were unable to secure the necessary instrument in London, and the patient was taken to a hospital with which a specialist, who was consulted, was associated. The guardians, beyond expressing the opinion that they were justified in calling attention to the fact that at two public hospitals a bed could not be found in a case of extreme urgency, have not as yet taken any steps to carry out a suggestion made some months ago that a special maternity ward should be set aside for paying patients.

### A BUILDING RACE AGAINST TIME.

Although originally opened in 1869, the Cottage Hospital, Stratton, North Cornwall, is virtually new on its reopening by Sir George Croydon Marks, M.P., after the completion of the extensions. The accommodation this month has risen from four to nine beds, including some for private patients; a tenth can be, a theatre has been, added and with the reconstruction of the original building the hospital now possesses an administrative block and a sanitary annexe. The sum of £4,000 has been spent on these alterations. The staff now consists of a matron, a nurse, and a probationer. And there is virtually no debt, thanks to the receipt in 1917 of two legacies, amounting to £1,500, under the wills of the late Misses Bray. It was a neck-and-neck race between the Ministry of Munitions and the hospital, for the wills provided that the new ward, for which the legacies were destined, must be completed within two years of the death of the surviving sister, and permission to build was granted by the Ministry only six months before the time-limit expired. Unusually sincere thanks are therefore due to the architect, Mr. B. C. Andrew, of St. Austell, and to the builders, Messrs. Pethick, of Bude, for materials were as difficult to obtain as time was short to use them.

### DISAPPEARING WORKHOUSES.

The reduction of the number of workhouses in Montgomeryshire to two is a noteworthy fact, in view of the action taken by the hospital authorities in Birmingham by co-operating with the Poor-law authorities there in regard to such buildings. It is a course which should receive full measure of consideration by the voluntary hospital authorities throughout the country. There is, at present, a serious shortage of hospital accommodation up and down the country, and before any existing buildings—workhouses or otherwise—can be made available for new objects and services, it is essential that the nature of such accommodation and its suitability or utility for hospital purposes or otherwise should be fully weighed and decided. Only then should any steps to close or demolish such buildings be entertained. The steps to close in Montgomeryshire have been taken in conjunction with the Ministry of Health, whereas in Glamorganshire, Cardiff has a waiting-list of 1,000 hospital patients. This indicates clearly that each county, in regard to hospital accommodation, must be considered separately in its own circumstances. For this reason the Ministry of Health would render substantial service to the hospitals throughout the country if they were to obtain a census of all unutilised Poor-law buildings, together with a report upon their suitability for hospital adaptation, and how far, if at all, such a step can be recommended. Failing this, to what purpose they can be otherwise utilised, with the greatest advantage to the Community and Nation.

### NO SHORT CUTS TO HOSPITAL SOLVENCY.

A letter signed Robert Vere Hodge has appeared in the *Birmingham Post* which suggests that the London Hospital has solved the problem of voluntary hospital finance by discarding the ticket system in favour of a request to each patient to contribute according to his means. Any hospital which discards the ticket system, which is nothing but petty patronage in its last ditch, is worthy of public gratitude; but it is too much to hope that patients' contributions, even if the rule, will suffice to solve the matter. We have heard no rumour that the London Hospital expects to be able to discard also its system of quinquennial appeals. Nevertheless, we note the writer's argument, partly because it is against the ticket system (which is an abomination), but chiefly because it shows how fond people are of a cut-and-dried solution. The true solution is not to be so lazily found. It is a scheme with brains as well as sympathy behind it, and one, moreover, which recognises that since hospitals are the friends of all classes, all classes must unite in their support. For this reason we have given a full-length description of the great piece of statesmanship which Mr. Braime has originated and described in detail for Leeds. Local conditions vary, but the principle and the method behind Mr. Braime's proposals are applicable in their degree to London as to Leeds, and to Birmingham as to London.

### CHILDREN'S HOSPITALS AND THE COLLEGE OF NURSING.

In three years' time the Irish Hospital for Sick Children, in Queen Street, Belfast, will celebrate its jubilee, which it is hoped will coincide with the erection of a new building. The committee are searching for a site, and the success of the plan entirely depends upon the support given by the public to it. The hospital, however, is devoting part of its energy to an attack upon the College of Nursing. It accuses the College of having made no recognition in its Registration Bill of Nurses who train in children's hospitals, and attacks the provision in the Bill which limits registration to those who have spent three years in a general hospital. The Hospital for Sick Children therefore has determined to join the Federation of Provincial and Children's Hospitals in order "to combat this injustice." Since, however, registration has been advocated because it gives to the nursing profession the stability accorded to doctors by similar enactment, it is strange that the method of the model should be objected to by the would-be imitators. No medical man is allowed by law to become a specialist until he has passed his general examination, nor can experience in one branch of study be set against ignorance of anatomy and physiology. Why, therefore, should nurses who wish to specialise—for the nursing of children is a speciality—be allowed registration without a general training? Hard cases will arise; but the law could deal gently with the elder women, while refusing registration after a recent date to any nurse whose training wholly takes place in a children's hospital.

### THE FIRST EFFECT OF MUNICIPALISATION.

The simplest arguments against rate-supported hospitals are those which are generally forgotten. We therefore congratulate Sir Hector Cameron on the old point he made when addressing lately the supporters of the Glasgow Eye Infirmary. Remember, he told them, that if hospitals are supported by the rates no one outside the rateable area will be admitted. The voluntary hospital refuses only those for whom it has no beds. The remedy, therefore, is to organise funds in the outlying districts, which often send patients instead of contributions.

### NEW SECRETARY OF ARUNDEL HOSPITAL.

Mr. J. A. Leggett, who consented to act for another year as honorary secretary of the Arundel Cottage Hospital, has now finally resigned. Mr. H. A. E. Hey has been appointed secretary to succeed him. By an alteration of an old rule the clergy are now eligible for election on the board of governors. It is odd that they should ever have been technically excluded, a fact due probably to the use of the word layman, which in hospital life usually means merely a man who is not a doctor.

### A CHAIRMAN AGAINST A GRANT IN AID.

An interesting discussion took place at a recent meeting of the Tottenham Urban District Council, when a proposal to give a grant in aid (on the basis of the value of 1d. rate) to the Prince of Wales's Hospital, Tottenham, was rejected. As it happened the chairman of the council, Mr. F. W. Jones, is also chairman of the Prince of Wales's Hospital Extension Fund. He opposed the grant in aid, because he believed it would be a cause of disaster to the hospital. The matter is a nice one, because no evil effect has followed similar grants in aid by local authorities when these have been given in return for special services, such as the treatment of school-children, or the like. Does a general grant in aid, apart from a particular service, make all the difference? We always prefer voluntary hospitals to finance themselves, but there are cases when Mr. Jones's argument does not apply. His opinion, in view of his double position on each body, is all the more interesting.

### THE EXETER HOSPITAL EXTENSION.

The foundation-stone of the new Victory Wing at the Royal Devon and Exeter Hospital has now been laid. The building is expected to cost £33,000, and to be ready next year, and will add 100 beds to the hospital. Sir Channing Wills and a grant from the Ministry of Pensions have made the scheme possible.

### AN IRISH SOLUTION OF HOSPITAL FINANCE.

The Irish Public Health Council has adopted an interesting report, drafted by its Chairman, Dr. Coey-Bigger, on the Irish hospital system. His main contention is that under various Acts of Parliament the local authorities have powers to send various classes of patients to voluntary hospitals, in which cases the State has agreed to pay from one-half to three-quarters of the cost. Local authorities, Dr. Coey-Bigger complains, have taken little advantage of these powers, "partly through apathy, and partly through ignorance." He mentions particularly the Tuberculosis Prevention Act of 1908, the Notification of Births Act (treatment for women during and after pregnancy), the Medical Treatment of Children Act, the Public Health (Prevention and Treatment of Diseases) Act of 1917 for venereal cases. Dr. Coey-Bigger argues that hospitals would benefit were these Acts used by local authorities, and that the duty of using them should be vested in one county authority. The voluntary system, he says, would be made solvent in this way.

### MEDICAL PREACHERS AT MANCHESTER.

Two distinguished Manchester surgeons, Sir William Milligan and Sir William Thorburn, have been setting an example which it is hoped will be followed generally. February 8 was "Hospital Sunday" in Manchester, and these two members of the honorary surgical staff of the Manchester Royal Infirmary filled the rôle of preacher on that day. In not a few places of worship "Hospital Sunday" does not receive that prominence which it deserves, and our institutions would ofttimes gain by the presence in the pulpit of some leading member of a board of management or professional staff well qualified by first-hand knowledge to win financial support. Sir William Milligan preached at St. Thomas's Church, Ardwick. He touched upon the question of nationalisation and showed what would be the dire results of bureaucratic control. So long as hospitals maintain their efficiency and are able to keep their heads above water no Government will interfere with a system which is the pride of the people. He urged the utilisation of Poor-law infirmaries by the Ministry of Health to increase accommodation, and advocated direct Labour representation upon voluntary hospital boards. Sir William Thorburn spoke at the Macfadyen Brotherhood, Chorlton-cum-Hardy, and said that from his experience of the almost total lack of the human touch in Government departments he was convinced that if the voluntary hospital system is abandoned we shall lose something of great value which we shall never regain.

### THIS WEEK'S DRUG MARKET.

High values are maintained all round, and one looks in vain for signs of any impending downward movement. Carbolic acid is substantially dearer. Citric acid is higher in price, and large supplies are difficult to find. Phenazone has an upward price tendency, but phenacetin is rather slow of sale at the moment. Hexamine is still in inadequate supply, while resorcin continues extremely scarce. Balsam Peru is dearer. Cascara sagrada is in fairly plentiful supply, and the price is not quite so firmly maintained. Cocoa butter is dearer. Nux vomica has an upward price tendency. Turkey opium seems likely to be rather dearer. The arrival of supplies of storax is worth noting. At the recent public sale of drugs in Mincing Lane good prices were paid for Cape aloes, and a high figure was paid for gum ammoniacum. Ipecacuanha sold at higher rates. Grey Jamaica sarsaparilla was dearer, as also were senna-leaves. Large quantities of honey were offered, but there was very little bidding. Gum guaiacum is rather lower in price, and calumba root, of which supplies are plentiful, seems likely to tend downwards in value.

